# *N*-Hydroxycinnamide Derivatives of Osthole Ameliorate Hyperglycemia through Activation of AMPK and p38 MAPK

**DOI:** 10.3390/molecules20034516

**Published:** 2015-03-11

**Authors:** Wei-Hwa Lee, Hsueh-Hsia Wu, Wei-Jan Huang, Yi-Ning Li, Ren-Jye Lin, Shyr-Yi Lin, Yu-Chih Liang

**Affiliations:** 1 Department of Pathology, Shuang Ho Hospital, Taipei Medical University, 291 Zhongzheng Rd., New Taipei City 23561, Taiwan; E-Mail: whlpath97616@shh.org.tw; 2 School of Medical Laboratory Science and Biotechnology, College of Medical Science and Technology, Taipei Medical University, 250 Wuxing St., Taipei 11031, Taiwan; E-Mails: wuhh@tmu.edu.tw (H.-H.W.); m120097071@tmu.edu.tw (Y.-N.L.); 3 Graduate Institute of Pharmacognosy Science, College of Pharmacy, Taipei Medical University, 250 Wuxing St., Taipei 11031, Taiwan; E-Mail: wjhuang@tmu.edu.tw; 4 Department of Primary Care Medicine, Taipei Medical University Hospital, 252 Wuxing St., Taipei 11031, Taiwan; E-Mails: linrenjye@tmu.edu.tw (R.-J.L.); sylin@tmu.edu.tw (S.-Y.L.); 5 Department of General Medicine, School of Medicine, College of Medicine, Taipei Medical University, 250 Wuxing St., Taipei 11031, Taiwan; 6 Traditional Herbal Medicine Research Center, Taipei Medical University Hospital, 252 Wuxing St., Taipei 11031, Taiwan

**Keywords:** osthole, *N*-hydroxycinnamide, AMP-activated protein kinase (AMPK), p38 MAPK, glucose uptake, skeletal muscle, streptozotocin

## Abstract

Our previous studies found that osthole markedly reduced blood glucose levels in both *db/db* and *ob/ob* mice. To improve the antidiabetic activity of osthole, a series of *N*-hydroxycinnamide derivatives of osthole were synthesized, and their hypoglycemia activities were examined *in vitro* and *in vivo*. Both *N*-hydroxycinnamide derivatives of osthole, OHC-4p and OHC-2m, had the greatest potential for activating AMPK and increasing glucose uptake by L6 skeletal muscle cells. In addition, OHC-4p and OHC-2m time- and dose-dependently increased phosphorylation levels of AMPK and p38 MAPK. The AMPK inhibitor, compound C, and the p38 MAPK inhibitor, SB203580, significantly reversed activation of AMPK and p38 MAPK, respectively, in OHC-4p- and OHC-2m-treated cells. Compound C and SB203580 also inhibited glucose uptake induced by OHC-4p and OHC-2m. Next, we found that OHC-4p and OHC-2m significantly increased glucose transporter 4 (GLUT4) translocation to plasma membranes and counteracted hyperglycemia in mice with streptozotocin-induced diabetes. These results suggest that activation of AMPK and p38 MAPK by OHC-4p and OHC-2m is associated with increased glucose uptake and GLUT4 translocation and subsequently led to amelioration of hyperglycemia. Therefore, OHC-4p and OHC-2m might have potential as antidiabetic agents for treating type 2 diabetes.

## 1. Introduction

AMP-activated protein kinase (AMPK), a serine/threonine kinase, plays a central role in regulating glucose and lipid metabolism and functions as a fuel gauge [1]. In skeletal muscles, the liver, and adipose tissues, activation of AMPK can increase metabolism, insulin sensitivity, and gene expression, and all of these effects are beneficial in preventing type 2 diabetes. Skeletal muscles have a primarily important role in maintaining normal glucose homeostasis, and type 2 diabetes is characterized by insulin resistance in skeletal muscles [2]. Glucose uptake is mainly mediated by glucose transporter 4 (GLUT4) and plays a crucial step in glucose utilization by insulin stimulation and muscle contractions [3]. Activation of phospatidylinositol-3 kinase (PI_3_K)/Akt and AMPK signal pathways, respectively, lead to increased insulin- and contraction-stimulated GLUT4 translocation in muscles [4]. Previous studies found that many antihyperglycemic chemicals can activate AMPK through different mechanisms, such as antidiabetic drugs, such as metformin [5] and troglitazone [6], and 5-aminoimidazole-4-carboxamide ribonucleoside (AICAR) [7], as well as natural polyphenols, such as resveratrol [8], curcumin [9], and epigallocatechin gallate [10].

p38 mitogen-activated protein kinase (MAPK) is a member of the MAPK family, and has wide-spectrum roles in controlling energy metabolism of adipose tissues, skeletal muscles, islet cells, and the liver [11]. Activation of p38 MAPK participates in stimulation of glucose uptake by both insulin and contraction stimuli in skeletal muscles [12]. As AMPK activators, many natural and synthesized chemicals are also able to increase glucose uptake and improve hyperglycemia through activation of p38 MAPK in different experimental models. In *db/db* diabetic mice, the synthesized compound, biaryl-4-carbonitrile, increased the phosphorylation of AMPK and p38 MAPK and ameliorated glucose uptake through the AMPK/p38 MAPK pathway [13]. The natural compound, capsaicin, also stimulated the phosphorylation of AMPK and p38 MAPK and subsequently increased glucose uptake by skeletal muscle cells [14].

Osthole is extracted from the Chinese herbs *Cnidium monnieri* and *Angelica pubescens*, and exhibits many biological activities such as antitumor [15], anti-inflammatory [16], antiosteoporosis [17], and antihyperlipidemic functions [18]. Recently, we found that osthole can ameliorate hyperglycemia in both *db/db* mice and mice with streptozocin (STZ)-induced diabetes, and the underlying mechanisms involved in these effects are associated with activation of AMPK and peroxisome proliferator-activated receptors α/γ (PPARα/γ) [19,20]. To improve the antihyperglycemic activity of osthole, OHC-4p and OHC-2m were semi-synthesized from osthole in this study. OHC-4p and OHC-2m were compared to osthole to evaluate their antihyperglycemic activities in skeletal muscle cells and understand the underlying molecular mechanisms.

## 2. Results and Discussion

### 2.1. OHC-4p and OHC-2m Were Superior to Osthole in Activating AMPK and Increasing Glucose Uptake in Skeletal Muscle Cells

**Figure 1:**
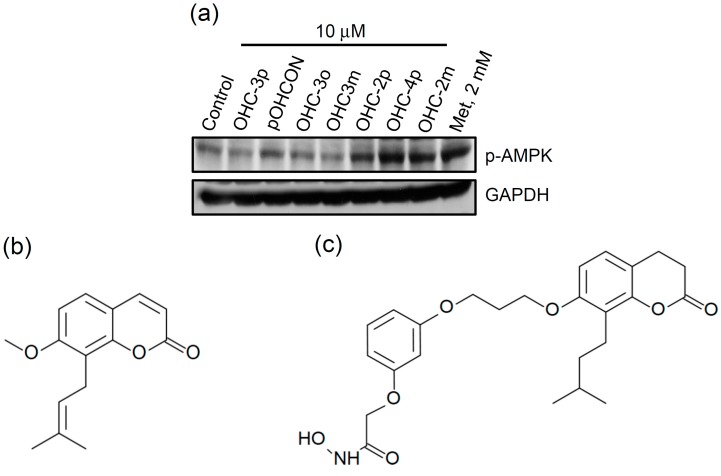

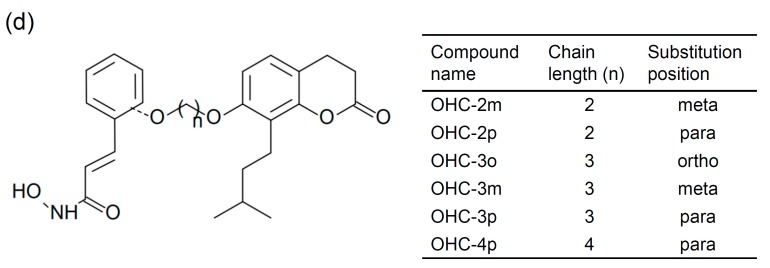
Screening of a series of *N*-hydroxycinnamide derivatives of osthole on the phosphorylation of AMPK and the chemical structures of (**b**) osthole and (**c**) osthole-derived phenyl-*N*-hydroxycarboxamate (pOHCON), and (**d**) various kinds of osthole-derived *N*-hydroxycinnamides. (**a**) Cells were treated with 10 µM of various kinds of osthole derivatives for 3 h. Total cell lysates were used to determine phosphorylation levels of AMPK by western blotting. Metformin (Met) was used as a positive control.

A series of *N*-hydroxycinnamide derivatives of osthole and a osthole-derived phenyl-*N*-hydroxycarboxamate (pOHCON) were synthesized (Figure 1b–d), and their ability to activate AMPK in differentiated L6 skeletal muscle cells was examined. As shown in Figure 1a, OHC-2p, OHC-4p, and OHC-2m exhibited greater abilities than the other osthole derivatives in increasing AMPK phosphorylation. The *N*-hydroxycinnamide had no any effect on the induction of AMPK phosphorylation (data not shown). Next, a pilot animal study was performed, and we found that OHC-4p and OHC-2m were able to improve hyperglycemia in mice with STZ-induced diabetes, while OHC-2p was not. Therefore, OHC-4p and OHC-2m were used in the subsequent experiments of this study. Compounds OHC-4p and OHC-2m were structurally characterized as *para*-osthole *N*-hydroxycinnamide with a chain-length of four carbons and *meta*-osthole *N*-hydroxycinnamide with a chain-length of two carbons, respectively (Figure 1d).

**Figure 2:**
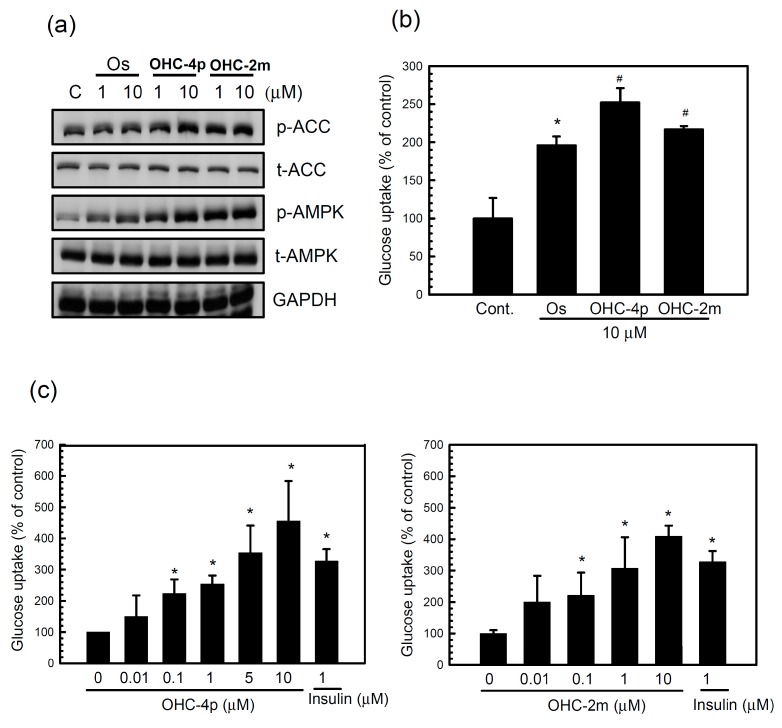
Comparisons of glucose uptake and AMP-activated protein kinase (AMPK) activation in differentiated L6 skeletal muscle cells between osthole and osthole derivatives. (**a**) Cells were treated with 1 or 10 µM of osthole, OHC-4p, or OHC-2m for 3 h. Total cell lysates were used to determine the phosphorylation levels of AMPK and acetyl-CoA carboxylase (ACC) by western blotting. (**b**,**c**) Cells were incubated with (b) 10 µM of osthole, OHC-4p, or OHC-2m for 3 h, and (c) various concentrations of OHC-4p or OHC-2m for 3 h, and then 2-NBDG uptake was determined as described in the Experimental Section. Data are presented as the mean ± S.D. of three independent experiments. * *p* < 0.05 *vs.* the control; ^#^ *p* < 0.05 *vs.* osthole treatment. Insulin (1 µM) was used as a positive control. p-ACC, phosphorylated ACC; p-AMPK, phosphorylated AMPK; t-ACC, total protein of ACC; t-AMPK, total protein of AMPK. Os, osthole.

Next, the abilities of OHC-4p and OHC-2m to activate AMPK and glucose uptake in differentiated L6 skeletal muscle cells were compared to the parental chemical, osthole. OHC-4p and OHC-2m produced greater increases in the phosphorylation of AMPK and its downstream mediator, acetyl-CoA carboxylase (ACC), as well as glucose uptake in differentiated L6 skeletal muscle cells compared to osthole (Figure 2a,b). Both OHC-4p and OHC-2m dose-dependently increased glucose uptake (Figure 2c). At 1~10 µM of OHC-4p and OHC-2m, we found no significant cytotoxicity during 24 h of treatment of differentiated L6 skeletal muscle cells. These results suggest that OHC-4p and OHC-2m are superior to osthole in activating AMPK and increasing glucose uptake by skeletal muscle cells.

### 2.2. OHC-4p and OHC-2m Stimulated Glucose Uptake that Depended on Activation of AMPK and p38 MAPK in Skeletal Muscle Cells

**Figure 3:**
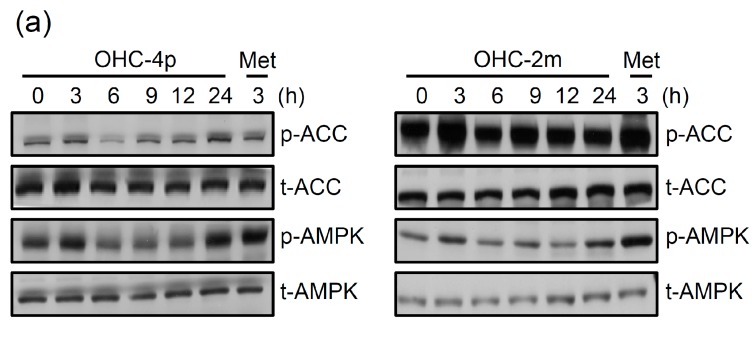

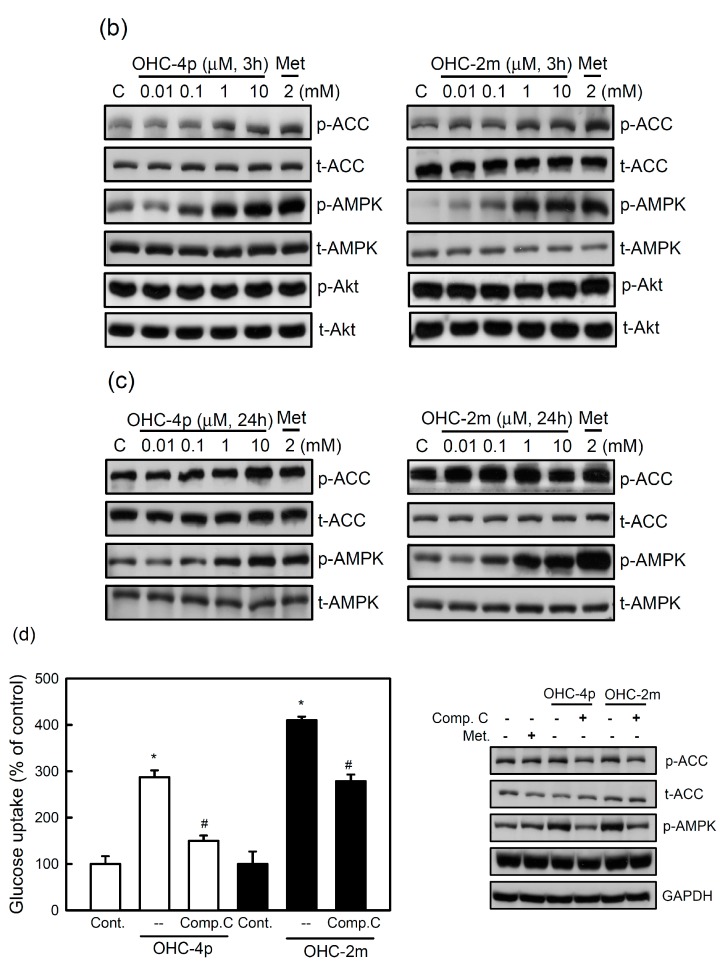
Involvement of AMP-activated protein kinase (AMPK) in glucose uptake induced by OHC-4p and OHC-2m in differentiated L6 skeletal muscle cells. (**a**) Cells were treated with 10 µM of OHC-4p or OHC-2m for different time periods; (**b**,**c**) Cells were treated with various concentrations of OHC-4p or OHC-2m for (b) 3 or (c) 24 h. Total cell lysates were used to determine the phosphorylation levels of AMPK and acetyl-CoA carboxylase (ACC) by western blotting. Metformin (Met) was used as a positive control. (**d**) Cells were treated with 10 µM OHC-4p or OHC-2m in the presence or absence of compound C (Comp. C, 10 µM) for 3 h and then 2-NBDG uptake was determined as described in the Experimental Section. Data are presented as the mean ± S.D. of three independent experiments. * *p* < 0.05 *vs.* the control; ^#^ *p* < 0.05 *vs.* OHC-4p alone or OHC-2m alone. p-ACC, phosphorylated ACC; p-AMPK, phosphorylated AMPK; t-ACC, total protein of ACC; t-AMPK, total protein of AMPK; p-Akt, phosphorylated Akt; t-Akt, total protein of Akt; Os, osthole.

It is known that activation of AMPK plays an important role in increasing glucose uptake [1]. OHC-4p and OHC-2m treatments significantly induced the phosphorylation of AMPK and ACC in differentiated L6 skeletal muscle cells (Figure 3a). Two maximal responses were seen at 3 and 24 h with both OHC-4p and OHC-2m treatments. There was no significant change in the AMPK phosphorylation during the culture period in the cells without drug treatment (Supplementary Figure S1). In addition, OHC-4p and OHC-2m treatments dose-dependently increased the phosphorylation of AMPK and ACC at 3 and 24 h (Figure 3b,c). To confirm the involvement of AMPK in OHC-4p- and OHC-2m-stimulated glucose uptake by L6 skeletal muscle cells, we treated cells with the selective AMPK inhibitor, compound C. As shown in the lower panel of Figure 3d, 10 µM compound C indeed decreased phosphorylation levels of AMPK and ACC in both OHC-4p- and OHC-2m-treated cells. Moreover, OHC-4p- and OHC-2m-induced glucose uptake levels were significantly reversed by compound C treatment (Figure 3d, lower panel).

**Figure 4:**
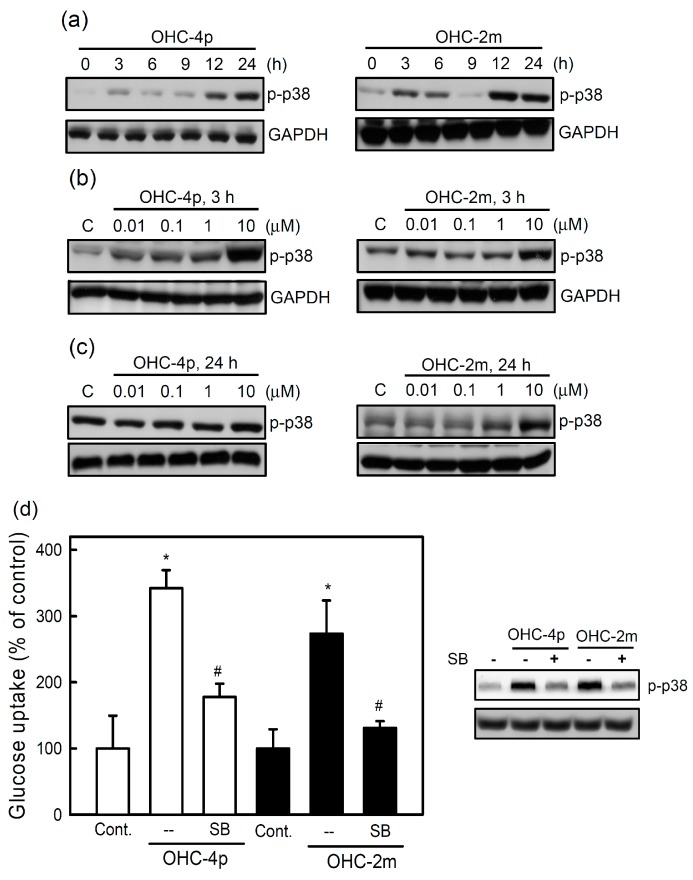
Involvement of p38 mitogen-activated protein kinase (MAPK) in the glucose uptake induced by OHC-4p and OHC-2m in differentiated L6 skeletal muscle cells. (**a**) Cells were treated with 10 µM of OHC-4p or OHC-2m for different time periods; (**b**,**c**) Cells were treated with various concentrations of OHC-4p and OHC-2m for (b) 3 or (c) 24 h. Total cell lysates were used to determine phosphorylation levels of p38 MAPK by western blotting. (**d**) Cells were treated with 10 µM OHC-4p or OHC-2m in the presence or absence of SB203580 (SB, 10 µM) for 3 h, and then 2-NBDG uptake was determined as described in the Experimental Section. Data are presented as the mean ± S.D. of three independent experiments. * *p* < 0.05 *vs.* the control; ^#^ *p* < 0.05 *vs.* OHC-4p alone or OHC-2m alone. p-p38 MAPK, phosphorylated p38 MAPK.

A previous study also demonstrated that p38 MAPK activation is involved in stimulating glucose uptake in response to insulin and muscle contractions [13]. We next investigated whether OHC-4p and OHC-2m stimulated glucose uptake through activation of p38 MAPK. After treatment with OHC-4p or OHC-2m, p38 MAPK phosphorylation reached a peak at 3 h and persisted from 12 to 24 h (Figure 4a). There was no significant change in the p38 phosphorylation during the culture period in the cells without drug treatment (Supplementary Figure S1). OHC-4p and OHC-2m also dose-dependently increased p38 MAPK phosphorylation at 3 and 24 h (Figure 4b,c). Moreover, OHC-4p- and OHC-2m-increased glucose uptake was inhibited by the p38 MAPK inhibitor, SB203580 (Figure 4d, lower panel).

**Figure 5:**
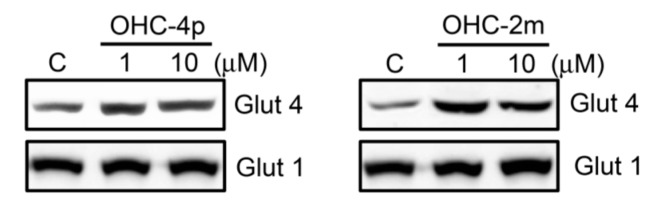
Effects of OHC-4p and OHC-2m on glucose transporter (GLUT) 4 translocation in differentiated L6 skeletal muscle cells. Cells were treated with 1 or 10 µM of OHC-4p or OHC-2m for 3 h. Equal amount of proteins (50 µg) from plasma membrane fractions were used to determine GLUT4 and GLUT1 (internal control) expression by western blotting.

As shown in Figure 5, 10 µM OHC-4p and OHC-2m caused about 3-fold increase in GLUT4 expression in the cell membrane fraction of differentiated L6 skeletal muscle cells. These results suggest that increased glucose uptake induced by OHC-4p and OHC-2m might be mediated through activation of both AMPK and p38 MAPK, and the subsequent increase in GLUT4 translocation in skeletal muscles.

### 2.3. OHC-4p and OHC-2m Improved Hyperglycemia in Mice with STZ-Induced Diabetes

**Figure 6:**
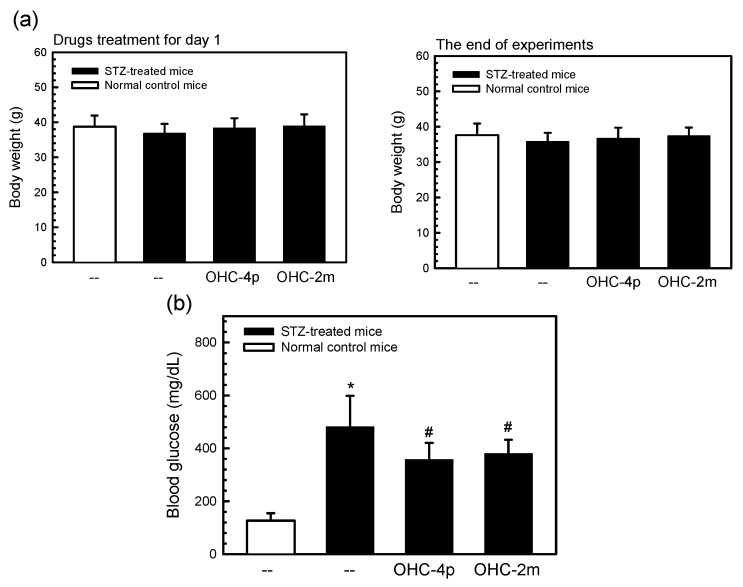
Effects of OHC-4p and OHC-2m on blood glucose levels of mice with streptozotocin (STZ)-induced diabetes mellitus. Diabetes was induced in male ICR mice (8 weeks old) by a single i.p. injection of 100 mg/kg STZ. Mice with STZ-induced diabetes were i.p. administered 20 mg/kg of OHC-4p or OHC-2m for 2 weeks. (**a**) The body weight was measured at the beginning and the end of drugs treatment; (**b**) Fasting blood was collected via heart puncture under pentobarbital anesthetization, and glucose levels were determined as described in the Experimental Section. Data are presented as the mean ± S.D. * *p* < 0.05 *vs.* the control; ^#^ *p* < 0.05 *vs.* STZ-treated mice.

To further prove the antidiabetic activities of OHC-4p and OHC-2m, we used mice with STZ-induced diabetes. In diabetic control mice, the fasting blood glucose level was about 480 mg/dL (Figure 6). However, OHC-4p and OHC-2m significantly decreased the blood glucose levels to 357 and 379 mg/dL, respectively, without affecting the body weight. The results suggest that OHC-4p and OHC-2m reduced blood glucose levels of mice with STZ-induced diabetes.

### 2.4. Discussion

Previous studies have reported that *N*-hydroxycinnamides exhibit several biological activities including antioxidant [21], anti-histone deacetylase (HDAC) [22,23], and anti-lipoxygenase properties [24]. We screened an in-house compound library containing *ortho*-, *para*-, and *meta*-substituted *N*-hydroxycinnamide derivatives of osthole for activation of AMPK in skeletal muscle cells and hypoglycemic activity in mice with STZ-induced diabetes, and found that both OHC-4p and OHC-2m showed potent activity. The aim of this study was to understand the underlying molecular mechanisms of OHC-4p and OHC-2m in ameliorating hyperglycemia, and which signaling pathways are involved. Our results demonstrated that OHC-4p and OHC-2m increased GLUT4 translocation and glucose uptake via activation of the AMPK and p38 MAPK signal pathways, and decreased blood glucose levels in mice with STZ-induced diabetes.

It is known that activation of AMPK and p38 MAPK can increase glucose uptake through increasing GLUT4 translocation to cell membranes. Besides these effects, several reports also found that activation of AMPK and p38 MAPK is able to upregulate GLUT4 gene expression following exercise and subsequently increase glucose uptake in skeletal muscles [25]. Myocyte enhancer factor 2 (MEF2) is a key transcription factor for GLUT4 gene expression and is inhibited by association with HDAC5 at rest [26]. During exercise, HDAC5 is phosphorylated by activated AMPK and results in dissociation from MEF2. Subsequently, MEF2 is phosphorylated by p38 MAPK and eventually combines with other coactivators to drive GLUT4 gene expression. OHC-4p and OHC-2m are potential HDAC inhibitors [22] and were found to activate both AMPK and p38 MAPK in this study. However, the mRNA and protein expressions of GLUT4 did not increase during 4 h treatment with OHC-4p or OHC-2m. The possibility that long-term treatment with OHC-4p and OHC-2m can increase GLUT4 gene expression in skeletal muscle cells, thus contributing to glucose uptake, cannot be ruled out.

Previous studies also indicated that p38 MAPK is a downstream component of the AMPK signaling pathway in AICAR-stimulated glucose transport [27] and is essential for maximal increases in glucose uptake by insulin treatment and contractions of skeletal muscle cells [12,28]. In this study, we found that both AMPK and p38 MAPK phosphorylation reached peaks at 3 and 24 h after OHC-4p and OHC-2m treatment (Figure 3 and Figure 4). These results suggest that p38 MAPK phosphorylation might be mediated by an AMPK-dependent pathway, and the first activation at 3 h and second activation at 24 h might respectively contribute to GLUT4 translocation to plasma membranes and GLUT4 gene expression. However, more experiments are still needed to support this supposition. The PI_3_K/Akt signal pathway is also involved in GLUT4 translocation to plasma membranes and glucose uptake by insulin-stimulated cells. Our previous studies demonstrated that osthole might stimulate glucose uptake through activation of Akt in skeletal muscle cells [20]. However, the current study demonstrated that neither osthole derivative, OHC-4p or OHC-2m, could increase Akt phosphorylation at 0.01–10 µM (Figure 3b). These results suggest that the increase of glucose uptake by OHC-4p and OHC-2m seemed to mainly be dependent on activation of AMPK and p38 MAPK, but be independent of the PI_3_K/Akt signal pathway.

A series of *N*-hydroxycinnamide derivatives of osthole was synthesized, and their anti-proliferative and antihyperglycemic activities were examined. WJ1376-1 (also named as OHC-3o) and WJ1398-1 (also named as OHC-4o) are *ortho*-substituted *N*-hydroxycinnamide derivatives of osthole, and were found to induce cell polyploidy and G_2_/M cell-cycle arrest in human colon adenocarcinoma cells through activating the ATR/Chk2 signaling pathway and inhibiting Aurora A kinase at a concentration of 15 µM [29]. OHC-4p and OHC-2m are *para*- and *meta*-substituted *N*-hydroxycinnamide derivatives of osthole, respectively, and contains a chain of an even number of carbons in length. However, <10 µM of OHC-4p or OHC-2m caused no toxicity toward skeletal muscle cells. In addition, *N*-hydroxycinnamide derivatives of osthole with even number of carbons chains (e.g., OHC-2p, OHC-2m, and OHC-4p) could increase the AMPK phosphorylation, but *N*-hydroxycinnamide derivatives of osthole with odd number of carbons chains (e.g., OHC-3o, OHC-3m, and OHC-3p) did not (Figure 1a). These results suggest that even and odd number carbon chains might influence the relative position between *N*-hydroxycinnamide group and osthole group, and this results in different abilities to activate AMPK. However, why *N*-hydroxycinnamide derivatives of osthole with different chain lengths and substituted positions have diverse biological functions needs to be investigated.

## 3. Experimental Section

### 3.1. Materials

Osthole was purchased from Wako Pure Chemical (Osaka, Japan). Osthole derivatives (Figure 1) were synthesized as previously described [22] and were estimated to be at least 95% pure by high-performance liquid chromatographic analysis. Both compounds were dissolved in sterile DMSO and stored at −20 °C. Metformin, compound C, and SB203580 were purchased from Tocris Bioscience (Ellisville, MO, USA). Insulin and a protease inhibitor cocktail were purchased from Sigma Chemical (St. Louis, MO, USA). 2-(*N*-(7-Nitrobenz-2-oxa-1,3-diazol-4-yl)amino)-2-deoxyglucose (2-NBDG) was purchased from Invitrogen Taiwan (Taipei, Taiwan). The anti-AMPK, anti-phospho-AMPK (Thr172), anti-ACC, anti-phospho-ACC (Ser79), anti-p38 MAPK, and anti-phospho-p38 MAPK antibodies were purchased from Cell Signaling Technology (Danvers, MA, USA). The anti-GLUT4 antibody was purchased from Santa Cruz Biotechnology (Santa Cruz, CA, USA), and the anti-GAPDH antibody was purchased from Abcam (Cambridge, MA, USA).

### 3.2. Cell Cultures and Myoblast Differentiation

Rat L6 skeletal myoblasts were kindly provided by Prof. Jim C. Fong (National Yang-Ming University, Taipei, Taiwan), and were cultured in Dulbecco’s modified Eagle medium (DMEM) containing 10% heat-inactivated fetal bovine serum (FBS). For myoblast differentiation, confluent myoblasts were incubated with differentiation medium (DMEM supplemented with 2% heat-inactivated horse serum) for 7 days, and the medium was refreshed every 3 days.

### 3.3. Cell Viability Assay

Differentiated L6 cells were treated with various concentrations of OHC-4p or OHC-2m for 24 h and then the cell viability was determined by a 3-(4,5)-dimethylthiahiazo(-z-y1)-3,5-diphenytetrazoliumromide (MTT) assay as described previously [29].

### 3.4. Preparation of Membrane Proteins and Western Blot Analysis

Briefly, cells were homogenized in a Dounce homogenizer with buffer A (20 mM Tris-C1 (pH 7.5), 2 mM EDTA, 0.5 mM EGTA, and a protease inhibitor cocktail). After brief centrifugation, the resulting supernatant was ultracentrifuged at 25,000 *g* for 1 h to obtain the pellet which represented the membrane fraction. The pellet was resuspended in buffer A containing 0.5% Triton X-100 and sonicated three times for 30 s each. After ultracentrifugation, the supernatant was collected as membrane proteins [20].

Thirty to fifty micrograms of protein was resolved by 10% or 12% sodium dodecylsulfate-polyacrylamide gel electrophoresis (SDS-PAGE) and transferred onto a polyvinylidene difluoride (PVDF) membrane (Millipore, Bedford, MA, USA) as described previously [20]. The PVDF membrane was then incubated with the following primary antibodies and subsequently incubated with an anti-mouse or anti-rabbit immunoglobulin G secondary antibody conjugated to horseradish peroxidase (Santa Cruz Biotechnology) and visualized using enhanced chemiluminescence kits (Santa Cruz Biotechnology).

### 3.5. Glucose Uptake Assay

Differentiated L6 cells were cultured in a 24-well plate. After drug treatment, cells were incubated with 50 µM 2-NBDG for 15 min, and then washed with phosphate-buffered saline (PBS) three times to remove any remaining 2-NBDG. The fluorescence intensity of cells containing 2-NBDG was measured on a Varioskan Flash spectral scanning multimode reader (Thermo Fisher Scientific, Waltham, MA, USA) with excitation at 485 nm and emission at 535 nm [20].

### 3.6. Induction of STZ-Induced Diabetes in Mice and Determination of Plasma Glucose Levels

Male ICR mice (8 weeks old) were purchased from the National Laboratory Animal Center (Taipei, Taiwan) and housed in an air-conditioned animal room. All animal experimental procedures were approved by the Institutional Animal Care and Use Committee of Taipei Medical University. Mice were starved 18 h before diabetes was induced with STZ, and then they received a single i.p. injection of 100 mg/kg of fresh STZ prepared in 0.05 M citrate buffer at pH 4.5 [20]. Three weeks after the STZ injection, the diabetic mice were divided into three groups (seven mice/group), and OHC-4p or OHC-2m was i.p. injected once every 2 days for 2 weeks. At the end of the experiment (week 8), fasting blood (starvation for 4 h) was collected via a heart puncture under pentobarbital anesthetization. The glucose level was measured using a GLUCOSE (GLUC-PAP) kit according the manufacturer’s manual (RANDOX Laboratories, Antrim, UK).

### 3.7. Statistical Analysis

Cell experimental data were analyzed by Student’s *t*-test, and animal experimental data were analyzed by a one-way analysis of variance (ANOVA). Data are expressed as the mean ± S.D., and differences were considered significant at *p* < 0.05.

## 4. Conclusions

Previous our studies have found that osthole exhibited antidiabetic activity in *db/db* and *ob/ob* mice. Here, we demonstrate that osthole derivatives OHC-4p and OHC-2m are better than osthole in the increase of glucose utilization in skeletal muscle cells. The OHC-4p and OHC-2m might have more potential than osthole to develop as therapeutic drugs for treating diabetes. 

## Supplementary Materials

Supplementary materials can be accessed at: http://www.mdpi.com/1420-3049/20/03/4516/s1.
