# Supplementary material for: N-Hydroxycinnamide Derivatives of Osthole Ameliorate Hyperglycemia through Activation of AMPK and p38 MAPK
Source: Molecules. 2015 Mar 11;20(3):4516–29. doi: 10.3390/molecules20034516 (PMC6272315; doi:10.3390/molecules20034516)
Supplement: Supplementary file 1 [file molecules-20-04516-s001.pdf]

Supplementary Material

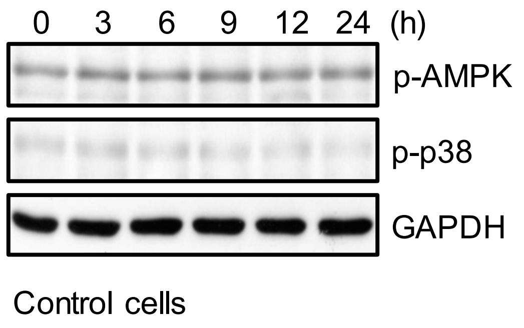

Figure S1. The phosphorylation levels of AMPK and p38 were constant during 24-h culture period in differentiated L6 skeletal muscle cells without drug treatment. Cells were seeded in culture dish for 24 h, and then continuously cultured for different time periods as indicated. Total cell lysates were used to determine the phosphorylation levels of AMPK and p38 by Western blotting. p-AMPK, phosphorylated AMPK; p-p38, phosphorylated p38.
